# Perceived depth reversals of images on a concave screen

**DOI:** 10.1177/20416695241249945

**Published:** 2024-05-08

**Authors:** Xiayi Gu, Han Yu, Hiroyuki Ito, Tama Kanematsu

**Affiliations:** Graduate School of Design, 12923 Kyushu University, Japan; Faculty of Design, 12923 Kyushu University, Japan;; Center for Applied Perceptual Science, Kyushu University, Japan

**Keywords:** perceived depth reversal, depth cues, reverspective, hollow mask illusion, binocular stereopsis

## Abstract

Reverspectives and hollow masks cause a reversal of perceived depth when observed from a position beyond certain critical distances, even if viewed binocularly. Their 3D structures or images invariably contain a linear perspective, shading, or familiarity cue to depth. Using a concave screen, we demonstrate a novel type of perceived depth reversal in binocular viewing with a variety of depth cues.

Reverspectives (Wade & Hughes, 1999), hollow masks (Gregory, 1970), and hollow dice (Rogers & Hughes, 2023) can reverse perceived depth even with binocular vision when they are viewed from a position beyond certain critical distances. However, they have limitations in terms of stimulus type. In reverspectives, an image is drawn with a perspective that is inversely correlated with the 3D structure of the canvas. The shape of a hollow mask is limited to a face. Using a concave screen, we demonstrate here that images containing convex depth cues can be perceived as convex against the binocular-disparity information indicating the screen concavity.

To confirm the effect of the 3D structure of a screen, we first observed 30 images projected onto a reverspective-type 3D structure. Three identical images were projected onto three horizontally arranged convex parts of the structure. Ten observers viewed them binocularly at a distance of 160 cm and reported the occurrence of perceived depth reversals on their first impression. The reversal rates were 98% for building images with linear perspective that was anticorrelated with the disparity information (i.e., reverspective), 61% for scenes of extensive landscape, 38% for building images with uncorrelated perspective, 26% for building images with correlated perspective, and 17% for face images. The reversal rates for plane images were 100% for uniform color images (red or green), 74% for low-contrast textures, 52% for high-contrast textures, and 27% for a black mesh image. We found that the reverspective-type 3D structure alone could induce the depth reversals because even face images projected onto the convex parts were sometimes reversed in depth, and because uniform color images caused perceived depth reversals at 100%. The contour of the reverspective-type 3D structure had a strong linear-perspective cue. The reversal rates were more or less dependent on the weakness of the image in masking the contour. Based on these initial observations, we then used a concave screen without visible contours inside.

A parabolic reflector (Edmund Optics, #80-254) was utilized as a concave screen, with its center hole covered with paper and its surface painted white (Figure 1 and Video 1). The images were presented using a focus-free laser-beam projector (AnyBeam, Pico Mini Portable Pocket Projector). The distance at which depth reversals occurred was measured as in Papathomas (2002).

**Figure 1.**
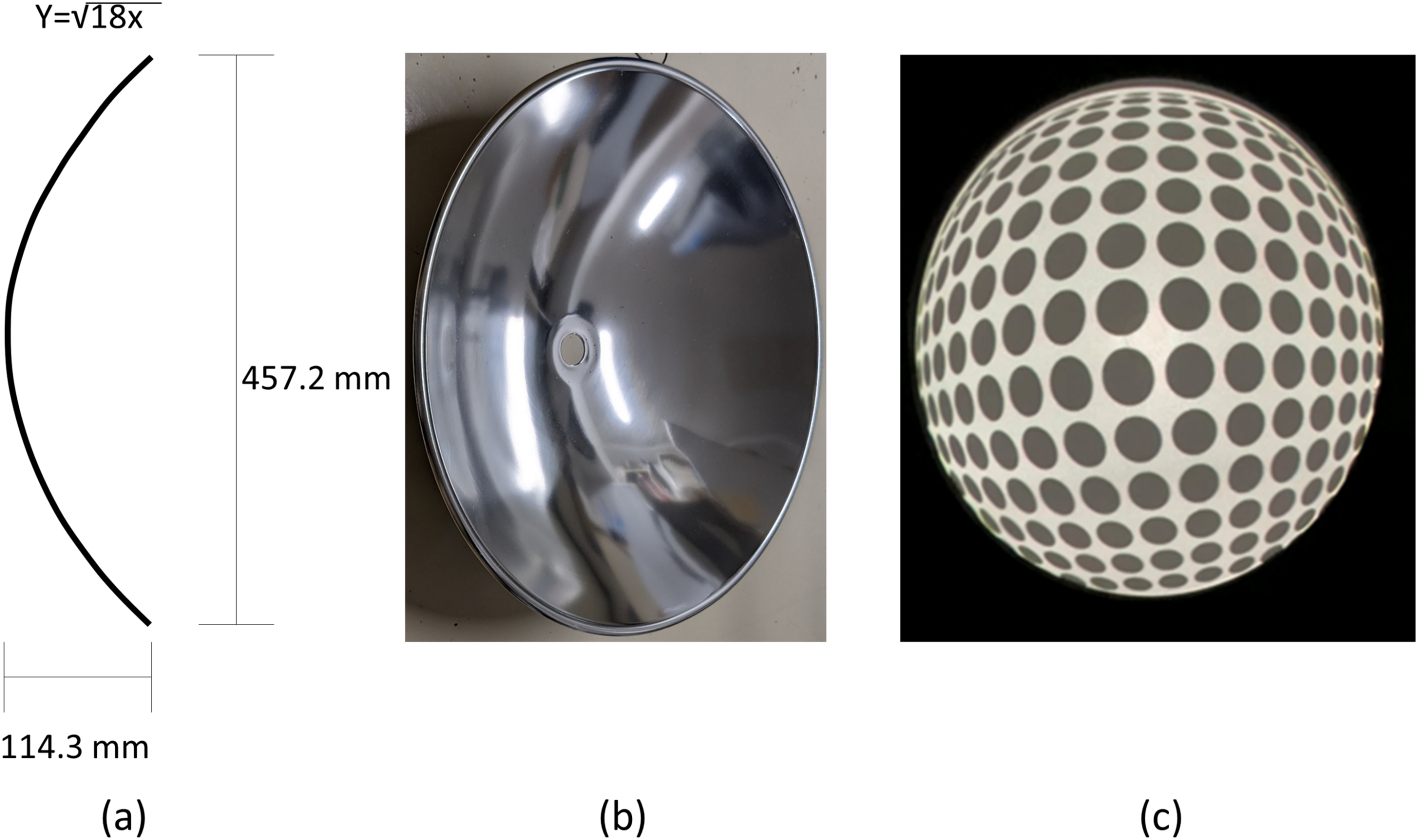
Concave screen. (a) Dimensions of the parabola shape. (b) Diagonal frontal view. (c) Sample of images projected on the screen.

**Video 1.** A sample stimulus.

Projected images are shown in Figure 2. A Method of Limits using both descending and ascending trials was used. In the descending series, the images were first viewed at a distance of 6 m; if they appeared to be convex or flat, the viewing distance was decreased by 50 cm, and the distance was recorded when the image perceptually changed to concave. In an ascending series, observers first viewed the image at a distance of 50 cm and then increased the distance by 50 cm. The images initially appeared as concave, but perceptually changed to flat or convex as the viewing distance increased. When the depth appearance changed, the distance was recorded. If the perceived concavity remained at a distance of 6 m, the critical distance was recorded as 6 m. Twelve observers each performed four binocular and four monocular observations. Half of the trials were performed with ascending series and the other half with descending series.

**Figure 2.**
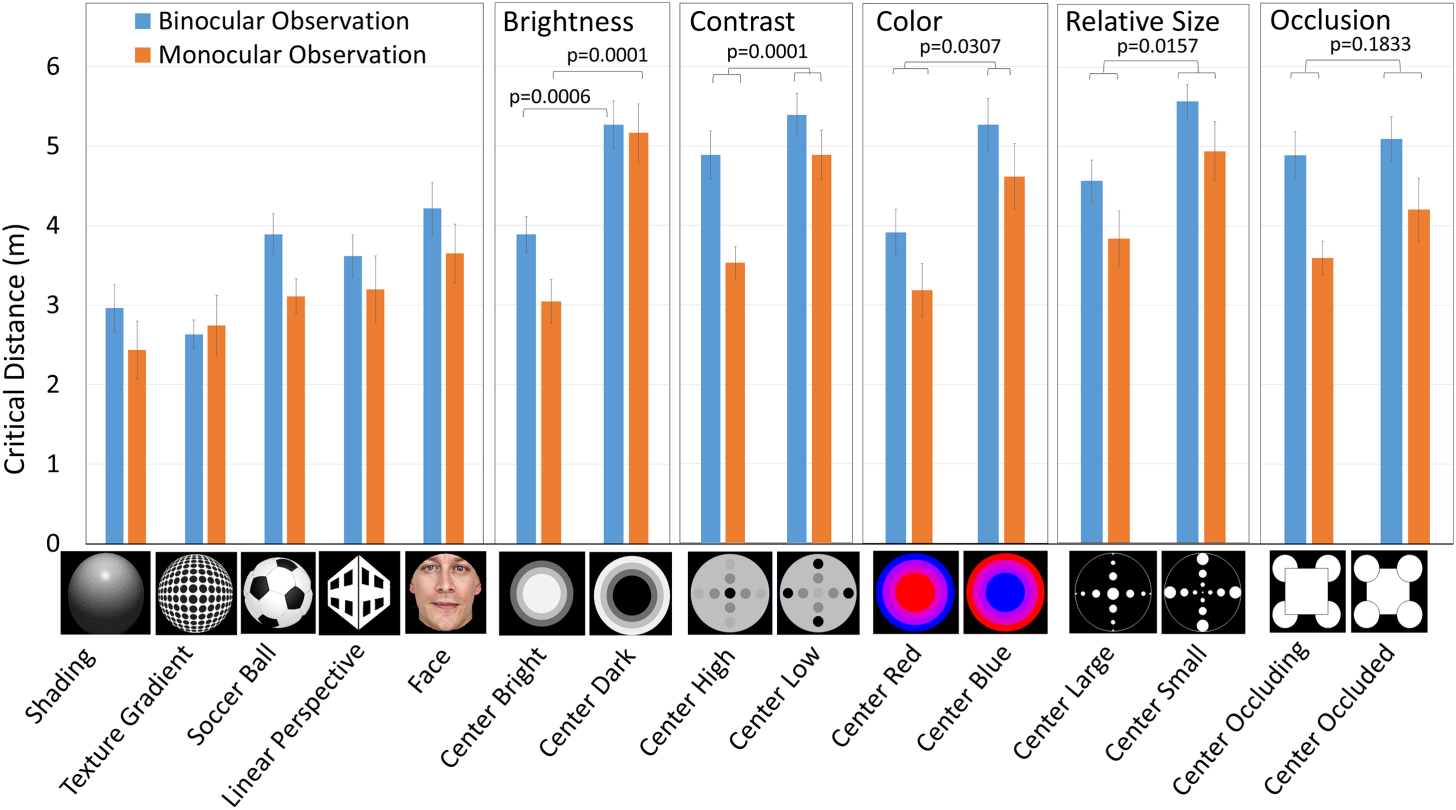
Critical distance at which perceptual concavity was broken.

Figure 2 shows the critical distance at which *concavity* was perceived as *flat* or *convex.* The critical distances in the ascending series were generally larger than those in the descending series, as is often reported. We used the averaged values. In particular, for the *texture-gradient* stimulus, all observers reported *convex* in all binocular trials at distances between 0.8 and 4.0 m. For the *shading* stimulus, all but one observers reported *convex* in all binocular trials at distances between 0.6 and 5.8 m. For the *soccer-ball* stimulus, all observers reported *flat* or *convex* in all binocular trials at distances between 1.5 and 6 m.

There are significant differences in critical distance between *center-bright* and *center-dark*, between *center-red* and *center-blue*, between *center-large* and *center-small*, and between *center-high* and *center-low* (in contrast) images. The critical distance for binocular viewing was significantly greater than for monocular viewing for most of the images.

Figure 3 shows that images with *shading*, *texture gradient*, *linear perspective*, and *familiarity* (*soccer ball*) cues indicating convexity were most effective in reversing perceived depth. These are mainly used in reverspectives or hollow masks. The *center-bright*, *center-red*, *center-large*, or *center-occluding* image elicited more *convex* responses than the *center-dark*, *center-blue*, *center-small*, or *center-occluded* image, respectively. These differences may reflect the depth suggested by each depth cue.

**Figure 3.**
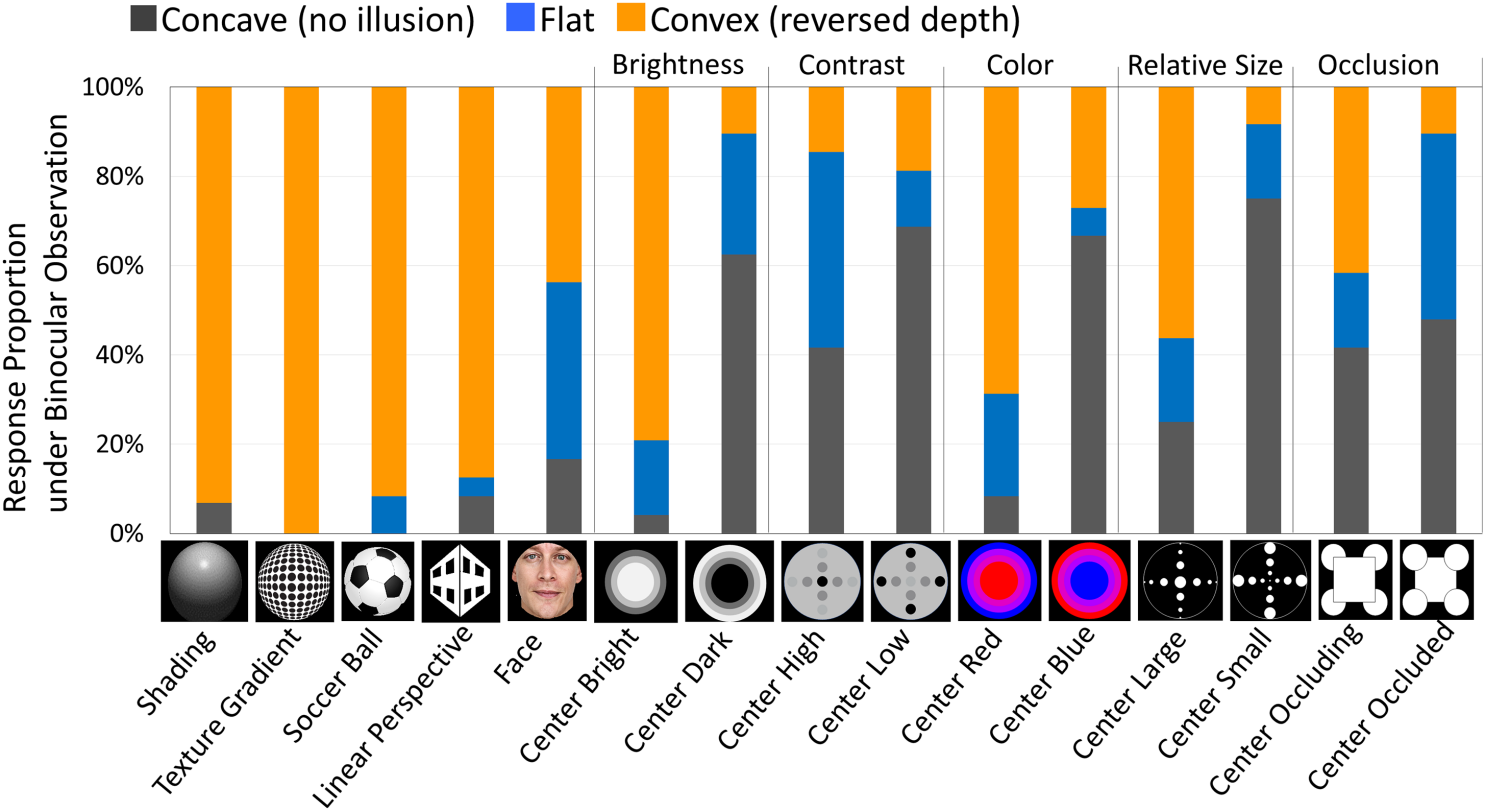
Reversal rates under binocular viewing. Orange bars indicate the percentage of trials in which *convex* (reversed depth) responses occurred. Blue bars indicate those of *flat* (but not *convex*) responses. Gray bars indicate those of *concave* responses (no illusion at any distance).

Compared to reverspectives and hollow masks, the advantage of our display is that it avoids the limitation of available images. There is no need for linear perspective or familiarity. The disadvantage is that a *linear-perspective* image or a *face* image is perceptually distorted by the movement of the observer. While a spherical shape image such as the *shading*, *texture-gradient,* or *soccer-ball* stimuli used here produced illusory motion during observer motion, the vertical contours in the *linear-perspective* image appeared to be bent by observer motion. The *face* image sometimes appeared to rotate unevenly, resulting in a perceived distortion of the face image. The perceived distortion sometimes breaks the perceived depth reversals.

We have demonstrated here a novel type of perceived depth reversal under binocular viewing (see also Piccolino and Wade (2011), describing a related illusion with a shadow of a church cross). The implication of the present results should not be closely tied to the physical concavity of the parabolic screen. As suggested by Rogers and Hughes (2023), with virtual-reality technology, one could create a variety of “reverspective” displays. Future experiments on depth-cue interactions will control binocular-disparity distributions independently of other depth cues. Recently, Wade and Hughes (2023) noted the properties of stereo pictures of reverspectives. To compare them with those of our displays would also be interesting.
